# The Influence of Socio-Affective Relationships Between Adolescents in Educational Experiences of Cooperation–Opposition: A Systematic Review

**DOI:** 10.3390/children12010015

**Published:** 2024-12-25

**Authors:** Paula Pla-Pla, Silvester Franchi, Pere Lavega-Burgués, Unai Sáez de Ocáriz

**Affiliations:** 1 Motor Action Research Group (GIAM), Institut de Desenvolupament Social i Territorial (INDEST), National Institute of Physical Education of Catalonia (INEFC), University of Barcelona (UB), Av. de l’Estadi 12-22, Anella Olímpica, E-08038 Barcelona, Spain; usaez@gencat.cat; 2 Motor Action Research Group (GIAM), University of West Santa Catarina (UNOESC), Joaçaba E-89600, Brazil; silvester.franchi@unoesc.edu.br; 3 Motor Action Research Group (GIAM), Institut de Desenvolupament Social i Territorial (INDEST), National Institute of Physical Education of Catalonia (INEFC), University of Lleida (UdL), Partida la Caparrella s/n, E-25192 Lleida, Spain; plavega@gencat.cat

**Keywords:** well-being, motor conflict, emotional dimension, relational dimension, motor conduct, physical education

## Abstract

Background/Objectives: Socio-affective relationships have garnered increasing attention in recent years as a means to enhance coexistence and well-being. Within this context, educational institutions play a pivotal role in shaping peaceful coexistence and promoting well-being among future generations. Physical Education (PE) is particularly significant, because it integrates cooperative–opposition activities, which blend collaboration and competition, fostering socio-emotional development. This systematic review aimed to investigate how PE contributes to coexistence and socio-affective well-being in adolescents aged 12 to 18. Methods: Using the PRISMA 2020 framework, 15 empirical studies were analyzed from seven databases. Studies were selected based on PICOS criteria: secondary education students (Population), cooperative–opposition activities (Intervention), control groups or pre-post designs (Comparison), relational and emotional dimensions (Outcomes), and quantitative methodologies (Study design). Results: Interventions grounded in the Motor Conduct Education and Sports Education Model significantly reduced interpersonal conflicts and improved social skills and emotional well-being. Positive emotions predominated in these activities, fostering stronger peer relationships within classroom groups. Approaches emphasizing task-oriented pedagogies were less effective than those centered on peer support in eliciting positive emotional responses. Conclusions: This review underscores the transformative potential of innovative educational strategies in PE to enhance coexistence and socio-affective well-being. Future research should explore the comparative efficacy of various pedagogical models and their long-term impact. These findings provide valuable guidance for educators and policymakers seeking to promote holistic development in adolescents through PE.

## 1. Introduction

One of the vital functions that distinguishes humans is the ability to relate socially and to experience emotions [1]. Life in society generates the need to establish bonds and relationships among individuals living together within a community [2]. Understanding the significance of social interactions and emotional connections is crucial for comprehending human behavior.

Coexistence, defined as the act of living together in mutual tolerance and respect, is more than just physical proximity; it involves a dynamic process of interaction, communication, and shared experiences [3,4]. It fosters an environment where individuals can learn from one another, develop empathy, and build trust [5,6,7]. Coexistence plays a fundamental role in shaping both individual and group personalities, emphasizing the interdependence among individuals [8].

Personality does not develop in isolation, but through continuous interaction with the social environment [9]. Thus, the social fabric of coexistence is integral to both individual development and the overall cohesion of society [3,5]. In the realm of community coexistence, interactions can give rise to harmonious agreements, fostering states of socio-affective well-being [10,11]. Conversely, diverging viewpoints and conflicting interests may precipitate tensions, leading to socio-affective discomfort and the potential emergence of interpersonal conflicts [12,13,14].

In this context, the ability to transform discomfort into well-being through effective management of relationships and emotions becomes crucial [15,16]. Promoting the transformation of tensions into opportunities for personal and collective growth is essential [5,17]. This involves equipping individuals with relational and emotional resources to facilitate peaceful conflict resolution [5,15,17,18]. Recent studies have shown that specific interventions can significantly reduce peer rejection, a key factor for academic and social success [7,19]. In this way, adversity can be turned into a source of learning that strengthens socio-affective well-being within the community, consolidating a more harmonious and resilient environment for coexistence [5,6,11].

Learning to live with others should form one of the foundational pillars of the education system, as endorsed by the Ministry of Education, Culture and Sport of Spain, along with European educational policies [20]. These esteemed institutions highlight the pivotal role of fostering interpersonal skills within contemporary education. Consequently, promoting school coexistence and enhancing well-being emerge as paramount imperatives within modern Physical Education [14,21]. Educational centers serve as optimal environments where children and adolescents engage in continual interaction, develop peer relationships, and share experiences [22,23,24,25]. These relationships become particularly significant during adolescence, a period marked by substantial personality changes and the development of individual and group identities [22,26].

For the sociologist Parlebas, Physical Education (PE) offers an ideal space for teaching coexistence, as students holistically activate the four dimensions of their personality through motor conduct (organic, decisional, relational, and emotional) [27,28]. It becomes evident that PE provides a unique platform for students to explore interpersonal dynamics and emotions through interaction [25,28,29,30,31,32]. Among the various educational tools available to PE educators, motor games emerge as particularly compelling pedagogical instruments for examining students’ interpersonal relationships [12,33,34,35]. This proposition is further supported by UNESCO’s Kazan Action Plan [21].

Motor games provide emotionally rich interpersonal experiences, shaping a small community that forms during play [36,37]. Within these games, rule agreements create a microcosm of society, wherein participants assume distinct roles and pursue varied objectives, fostering an environment where coexistence—including gender dynamics—is a valuable learning outcome [27]. The emphasis on cooperation–opposition scenarios enhances interactions among participants by facilitating relationships with both teammates and opponents [12,13,30,33].

Previous literature reviews on the connection between interpersonal relationships and emotions within educational and physical activity contexts suggest that socio-affective relationships play a fundamental role in improving students’ attitudes and behaviors towards themselves and others [38,39,40,41,42,43]. This highlights an interesting area for further exploration, integrating interpersonal relationships with the study of emotions.

Despite the acknowledged formative role of cooperative–oppositional games in PE and their potential to shape socio-affective development, a deeper analysis is required to fully comprehend the underlying mechanisms that make these games effective. Existing studies often highlight the positive impact of these games in improving interpersonal relationships and emotional competencies. However, the specific processes through which these outcomes are achieved remain insufficiently explored. For instance, while the general benefits of these interventions are acknowledged, there is a lack of clarity on how different pedagogical models influence the development of emotional regulation and empathy among adolescents in Physical Education contexts [12,13,33,44]. Moreover, most research has focused on short-term impacts, with little attention given to the long-term effects of such interventions on the socio-affective well-being of students. Longitudinal studies are needed to better understand how these experiences evolve over time and whether they lead to sustained changes in students’ relational and emotional competencies.

Additionally, although studies have explored socio-affective outcomes in PE, many fail to consider the contextual variables that might influence the effectiveness of these interventions, such as the cultural background of students, their previous experiences with conflict, and the specific teaching styles employed. This gap in the literature suggests that the role of educators, their pedagogical approaches, and the environmental factors within schools should be examined more closely, to understand how these elements interact with the game-based learning processes.

The present research aims to address this gap by conducting a comprehensive systematic review of the literature. By examining the relationship between cooperative–oppositional situations in PE and the development of socio-affective relationships, this study seeks to illuminate the underlying mechanisms driving this process. Furthermore, it underscores the importance of addressing this topic through a rigorous, evidence-based approach, synthesizing and critically analyzing prior empirical studies. This research aims to contribute to advancing knowledge in PE and developmental psychology, while offering valuable insights for educators, researchers, and professionals interested in promoting socio-affective well-being in adolescents, through educational interventions rooted in cooperative–oppositional motor games.

The primary objective of this study is to systematically review the existing literature to identify educational strategies within Physical Education that foster socio-affective relationships through cooperative–opposition situations among adolescents aged 12 to 18.

## 2. Materials and Methods

The design of this study corresponds to a systematic review, in order to investigate the socio-affective impact on interpersonal relationships arising from varied pedagogical experiences based on motor games or cooperation-opposition sports in formal education. The review protocol was developed in accordance with the Preferred Reporting Items for Systematic Reviews and Meta-Analyses (PRISMA) guidelines [45]; the PRISMA checklist can be found in Table S1.

### 2.1. Systematic Review Protocol

The protocol of this study and the data supporting the findings are available at the Open Science Framework (OSF; https://osf.io/ha5fd, accessed on 21 December 2024).

### 2.2. Search Strategy

The search was carried out in seven electronic databases with studies published until December 2023: PubMed, SPORTDiscus, Scopus, Web of Science, ERIC, Medline and SciELO. To ensure a focused approach, the search strategy was limited to open-access documents available within these databases, and restricted to studies published in Spanish, Catalan, or French. The search terms combined four main search terms and the Boolean operator “AND”, as follows:

(“Formal education” OR “high-school” OR “Physical education” OR Secondary OR adolescent* OR teen* OR Student) AND (“Motor games” OR cooperation–opposition OR “Collective sport” OR “Invasion sport*” OR “Invasion game*” OR “Traditional Sporting Games”) AND (Sociometr* OR Sociogram* OR Coexistence OR “Interpersonal relation*” OR “social interaction*” OR Well-being OR socio-affective* OR “socio affect*” OR socioaffect* OR “Motor conflict*” OR Emotion* OR “motor conduct” OR Conflict* OR “interpersonal conflict*” OR Behavi#r* OR “disruptive behavi#r*” OR “violent behavi#r*” OR “aggressive behavi#r *” OR “motor conduct*” OR “motor praxeology*” OR “conflict behav#r*” OR “sport behavioral behav#r*” OR “emotional dimension” OR “relational dimension” OR “affective dimension” OR “social dimension ” OR “social relation”) AND (“methodology” or “education method” OR “education” OR “pedagogy intervention” OR “intervention” OR pedagogy).

These terms were systematically applied to the keyword, title, and abstract fields, to ensure the inclusion of all relevant studies meeting the specified criteria.

### 2.3. Inclusion Criteria

The inclusion criteria were formulated using the PICOS framework:

- Population: compulsory secondary-education students.
- Intervention: driving situations of cooperation–opposition.
- Comparison: for studies without a control group, those comparing outcomes using pre- and post-tests or prospectively collected data across sessions of an educational learning situation.
- Results: quantitative measures on the emotional and relational dimension.
- Study typology: designs limited to quantitative studies, including randomized clinical trials, quasi-experimental designs, and observational studies.

### 2.4. Exclusion Criteria

Narrative reviews, systematic reviews and meta-analyses, non-experimental studies, proposed study protocols, theses and conference abstracts or proceedings were excluded.

#### 2.4.1. Study Selection, Data Extraction and Study Quality

The search results from various databases were imported into Rayyan systematic review software [46], where duplicate entries were identified and removed. Subsequently, the inclusion and exclusion process was conducted using a double-blind approach, with two reviewers independently assessing the studies. Any discrepancies were resolved through consensus between the two reviewers. Initially, the screening phase involved evaluating the titles and abstracts of the studies, followed by a detailed assessment of the full texts during the eligibility phase. Additionally, the reference lists of included studies were manually examined to identify any additional eligible studies, which were subjected to the same screening procedure.

The reviewers also performed a quality assessment using the risk of bias in non-randomized intervention studies (ROBINS-I) tool [47]. Potential bias factors included confounding variables, participant selection, intervention classification, deviations from planned interventions, missing data, outcome measurement, and reporting selection. An overall risk level was determined, following the instrument’s protocol.

To comply with PRISMA 2020, the study characteristics and outcomes were summarized in a table (Table 1). This table provides detailed information on the study design, population size, intervention types, measured outcomes, and main findings.

#### 2.4.2. Data Analysis

Due to the high level of heterogeneity among assessment instruments used for pedagogical interventions in the included studies, statistical grouping was deemed inappropriate. Instead, data synthesis was performed narratively, supported by tables presenting the corresponding data. In this study, we decided not to conduct a meta-analysis due to the significant heterogeneity across the included studies, both in terms of methodological design and the interventions evaluated. Variations in the approaches to Physical Education programs, sample characteristics, implementation contexts, and measurement tools used made it challenging to combine the results into a single quantitative effect size. Such variability could introduce bias or misinterpretations if a meta-analysis were conducted. Instead, we adopted a narrative synthesis approach, which allowed us to examine and discuss the findings in their original context, providing a more nuanced understanding of the socio-affective relationships in Physical Education.

## 3. Results

### 3.1. Studies Included

**Figure 1:**
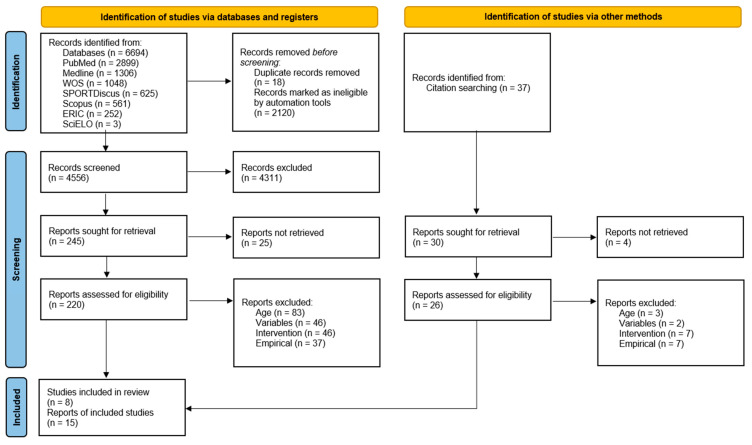
PRISMA flow diagram illustrating the study search process.

A PRISMA flowchart [45] detailing the complete search process is presented below (Figure 1). A total of 6694 references were identified across electronic databases. After removing duplicates (*n* = 2138), 4311 studies were excluded during the title and abstract screening phase. Following full-text analysis and the application of exclusion criteria, 11 articles were included. Additionally, a manual search of reference lists yielded 37 references, of which 8 studies met the inclusion criteria. In total, 15 studies were selected for this systematic review.

To ensure inter-rater reliability, the Kappa coefficient was calculated [59], evaluating the relevance of the selection criteria. Inter-rater reliability scores were *k*= 0.99 for title and abstract screening, and *k* = 1.00 for full-text reviews, resulting in an overall Kappa coefficient of *k* = 1.00. These findings indicate perfect agreement between reviewers, affirming the reliability of the selection process.

### 3.2. Characteristics of the Studies

The reviewed studies included 14 quasi-experimental designs and 1 randomized controlled trial (RCT). Quasi-experimental studies were further categorized into a pre-post single group with repeated measures (*n* = 8) and a pre-post with control group (*n* = 6). All studies were conducted within secondary education settings, and included participants aged 12 to 18 years.

**Figure 2:**
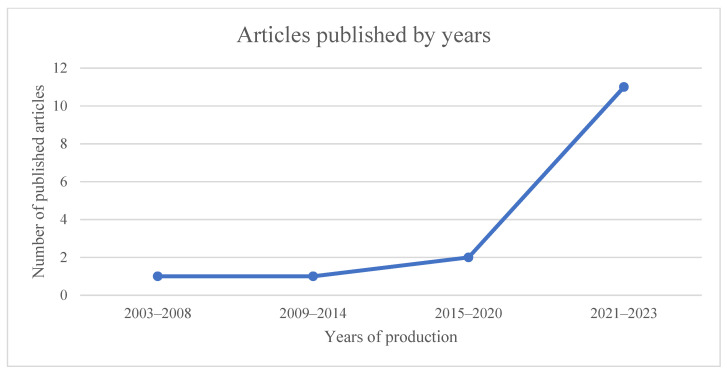
Number of articles published by year.

**Figure 3:**
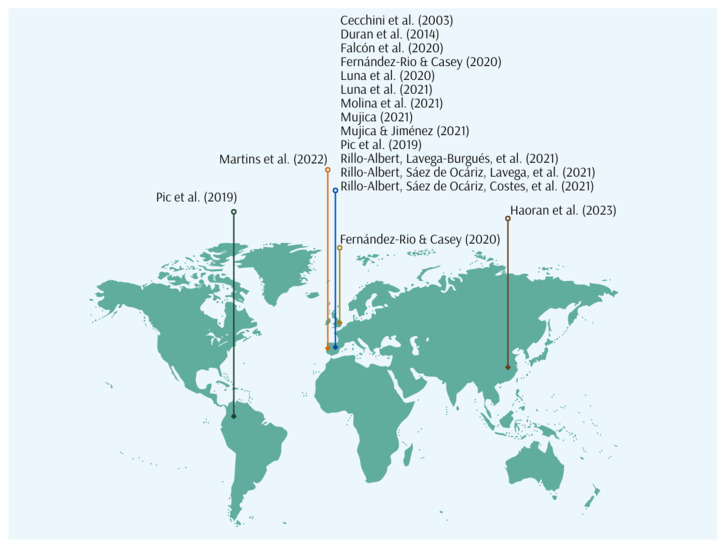
Geographical location of the countries of origin of the publications [12,13,33,44,48,49,50,51,52,53,54,55,56,57,58].

The studies were published between 2003 and 2023 (Figure 2), spanning five countries (Figure 3): Spain (*n* = 13), Portugal (*n* = 1), the UK (*n* = 1), China (*n* = 1) and Colombia (*n* = 1).

### 3.3. Nature of the Pedagogical Intervention

The pedagogical approaches utilized in the included studies encompassed cooperation–opposition sports (basketball, soccer, and futsal) (*n* = 7), motor games or traditional sporting games (*n* = 6), and split-team or net sports (*n* = 2). The educational methodologies implemented in the experimental groups (EG) were as follows: Teaching Personal and Social Responsibility (TPSR) (*n* = 2), Motor Conduct Education (MCE) (*n* = 3), Sports Education Model (SEM) (*n* = 3), and unspecified approaches in the remaining cases (*n* = 7). For the control groups (CG), the traditional methodology was applied using direct instruction (*n* = 3), competition was suppressed (*n* = 1), and no intervention was implemented in the others (*n* = 2).

The studies assessed the emotional dimension using the Games and Emotions Scales (GES) (*n* = 3), the Games and Emotions Scales II (GES-II) (*n* = 2), semi-structured interviews and personal diaries (*n* = 2), and the Motivational Climate of Peers in Sport Scale (PeerMCYSQ) (*n* = 3). To evaluate the relational dimension, the following tools were used: the Motor Conflict Questionnaire (MCQ) (*n* = 1), Adolescent Multidimensional Social Competence Questionnaire (AMSC-Q) (*n* = 1), Fair Play Attitude Scale (OFC) (*n* = 1), Interpersonal Relationship Scale (FIAT–Q) (*n* = 1), Observation of Fair Play Behaviors (*n* = 1), Personal and Social Responsibility Questionnaire (PSRQ) (*n* = 1), Cooperative Learning Questionnaire (*n* = 1), Child and Adolescent Self-Control Questionnaire (CACIA) (*n* = 1), Self-Efficacy Inventory for Multiple Intelligences (IAMI) (*n* = 1), and the Social Adjustment Scale for Children and Adolescents (*n* = 1).

### 3.4. Methodological Quality

**Figure 4:**
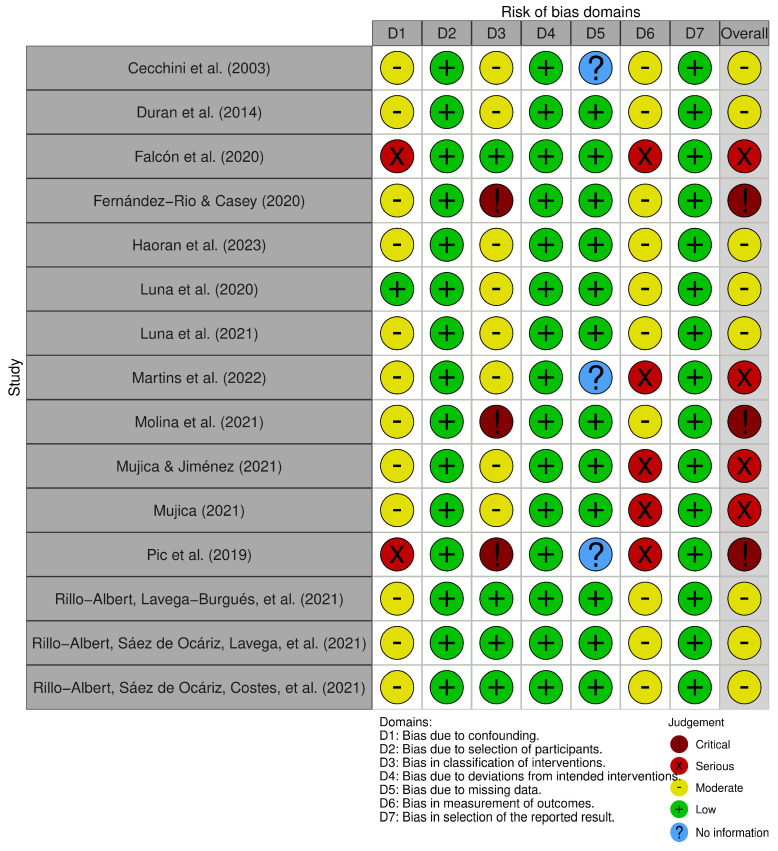
Risk of bias assessment with the ROBINS-I tool [12,13,33,44,48,49,50,51,52,53,54,55,56,57,58].

The ROBINS-I tool [47] revealed that the majority of articles were at moderate (*n* = 8; 53%), serious (*n* = 4; 27%) and critical (*n* = 3; 20%) overall risk of bias (Figure 4). Studies with a high risk of bias come from the possible effects of confounding and in the measurement of the results. However, all studies followed the intended intervention. Furthermore, bias in the domain of participant selection and selection of reported outcomes was mainly low.

### 3.5. Experimental Groups

#### 3.5.1. Effect on the Relational Dimension

The interventions consistently led to significant improvements in interpersonal relationships among participants [12,13,33,44,50,51,52,54]. These changes included a reduction in motor tensions and conflicts [12,13,33], and an increase in positive relationships and attitudes [13,44,50,52]. Improvements in classroom atmosphere enhanced participants’ personal and social responsibility [51,52], fostering efficacy and satisfaction and contributing to a positive group dynamic during Physical Education sessions [12,44,52].

#### 3.5.2. Effect on the Emotional Dimension

The emotional outcomes demonstrated a predominance of positive emotions over negative and neutral ones [12,48,49,58]. Regarding gender differences, girls tended to associate boredom with practices with a technical goal, while boys showed positive emotions in situations linked to motor competence and sports performance [56,57].

The pedagogical approach significantly influenced emotional responses. Task-oriented educational activities often triggered negative emotions, whereas peer-supported methods fostered positive emotions [13,33,56,57]. These findings highlight the importance of fostering a supportive classroom environment and quality social interactions in PE settings [13,33,57].

#### 3.5.3. Control Groups

Six of the articles included in the study utilized a control group to facilitate result comparison. Among these, three articles opted for the Traditional Direct Instruction Model (TM-DI) methodology [50,52,53]. This approach, characterized by its conventional pedagogical structure and focus on direct instruction, served as a benchmark to assess the implementation of alternative strategies or models, enabling a comparison of observed outcomes and perceived benefits.

During the initial phase of data collection, both the control group (CG) and experimental group (EG) were initially deemed homogeneous [50,52,53]. However, upon completion of the intervention program, significant discrepancies emerged between participants exposed to the experimental and control conditions, with the EG demonstrating notably higher scores on the relational dimension [50,52,53].

In other articles where the teacher-led programming was followed, multivariate analysis results confirmed substantial changes between the CG and EG, with decreases observed in anti-regulatory behaviors and improvements in personal and social responsibility [54,59]. These changes were not evident in the CG, further affirming that the observed effects can be attributed to the educational program intervention.

In the study where the competitive phase was omitted, results indicated a positive influence on interpersonal relationships, personal identity, and social adjustment among high school students [51]. This outcome underscores the significance of considering the impacts of various intervention methodologies on student development.

#### 3.5.4. Comparison of Different Learning Methodologies

Eight reviewed studies have implemented in the EG various pedagogical approaches in motor situations of cooperation–opposition in adolescents. Two of these studies used the Teaching of Personal and Social Responsibility (TPSR) methodology, and focused attention on the relational dimension of PE, with the aim of promoting the development of personal and social responsibility among students. The findings highlighted significant improvements in variables associated with personal and social responsibility within the experimental group (EG), accompanied by enhancements in ethical and prosocial behaviors [54,59].

In three studies employing the MCE, findings indicated a reduction in motor conflicts, enhanced task atmosphere scores, intensified positive emotions, and improved interpersonal relationships among students [12,13,33].

Finally, three studies implemented SEM as a pedagogical approach, wherein an increase in students’ interpersonal skills was observed, along with improvements in prosocial behavior and cooperation [52]. Additionally, a significant enhancement in the relationships among participants in the experimental group was highlighted [50,53].

## 4. Discussion

This study aimed to investigate the impact of educational interventions on interpersonal relationships, emotional states, and overall socio-affective development among adolescents in educational settings. Through a comprehensive analysis of the reviewed literature, this discussion elucidates the key findings and implications of these interventions in promoting positive socio-emotional outcomes among adolescents.

### 4.1. Positive Effect on the Relational Dimension

The reviewed studies consistently agree on a key finding: specific interventions reduce motor conflicts, thereby decreasing tensions and confrontations, while improving interpersonal relationships [12,13,44,50,51,52,53,54,59]. These findings align with those of Opstoel et al. [41], who concluded that collaborative work in PE classrooms fosters personal and social development among students. Moreover, creating a positive educational climate was associated with outcomes such as increased perceived efficacy and prosocial behaviors [51,52] findings, similar to those reported in the systematic review by Martín-Villar et al. [40]. These results highlight the transformative potential of interventions in fostering cohesive and cooperative group dynamics.

### 4.2. Influence of Interventions on Emotional States

A literature review on emotions in PE revealed several aspects in relation to the influence of these emotions on student learning and participation in this subject [60]. Physical Education interventions have demonstrated a positive effect on students’ emotional states, although there is often an imbalance in emotional intensity [12,13,33,48,49,55,56,57,58]. Taking into account other systematic reviews on emotions in PE, it is agreed that negative emotions are predictors of conflict and disruptive attitudes [42,43] whereas positive emotions contribute to fostering good interpersonal relationships [39]. Emotional preferences exhibit variations based on gender, underscoring the significance of forming heterogeneous groups in educational activities [56,57]. The increase in positive emotions affects the learning atmosphere, highlighting the imperative for more comprehensive and innovative practices [13,33,56,57].

### 4.3. The Impact of Pedagogical Interventions on Socio-Affectiveness

The studies that have been reviewed have applied various pedagogical methodologies in cooperation–opposition situations between adolescents. The positive effects of methodologies focused on personal and social responsibility, and ECM and SEM are highlighted [12,13,33,50,52,53,54,59]. The systematic review of Hovdal et al. [61] states that the methodologies focused on personal and social responsibility yield more positive outcomes in conflict education. Studies with CG reinforce the importance of evaluating the effects of different models on student development [50,51,52,53,54,59]. The ECM emerges as a powerful tool to enhance both the coexistence and the socio-affectiveness of students, contributing integrally to the educational environment [12,13,33]. Sport Education (SEM) and Motor Conduct Education (ECM) are effective pedagogical methodologies used in Physical Education (PE) to foster socio-emotional and behavioral outcomes in students. Understanding how these outcomes are activated and reinforced within the context of PE is crucial to comprehending their full impact.

Sport Education is a methodology that aims to replicate the structure of competitive sports within the classroom, where students take on various roles such as athletes, coaches, referees, and team managers [62]. This approach focuses on not only developing physical skills, but also enhancing social and emotional competencies by providing students with authentic, active learning experiences [63]. By participating in extended sport seasons, students engage in a series of progressive challenges that require teamwork, leadership, and self-regulation [63]. The outcomes of Sport Education—such as increased cooperation, communication, and problem-solving skills—are activated through the sustained practice of these roles and responsibilities within the sports setting [62]. As students take ownership of their roles, they internalize values such as fairness, respect, and empathy, which are continuously reinforced throughout the sport season [62,63,64]. These values and social skills, which are developed within the PE setting, extend beyond the classroom, contributing to broader socio-emotional development in other contexts.

On the other hand, Motor Conduct Education (MCE) conceptualizes each student as a unique individual who applies all aspects of their personality (organic, relational, emotional, and cognitive dimensions) to respond in a unique way to the demands of any motor situation [30,65]. This holistic perspective goes beyond simply performing exercises; it emphasizes the development of the entire person [30]. The aim is to optimize the students’ multidimensional development, influencing their personal, social, and physical growth [30,66]. Thus, through reflection in/for and through action, it creates an educational space where students are not only practicing motor skills but are also engaging in continuous dialogue about their emotional and social behaviors [65,66]. This process fosters the development of critical thinking, self-regulation, emotional intelligence, and effective communication—key components of socio-emotional well-being [30]. By integrating this reflective practice into motor activities, MCE supports the holistic development of students, enabling them to become more self-aware and responsible individuals.

These findings can serve as a foundation for the design and implementation of specific strategies aimed at promoting the socio-affective well-being of adolescents in educational settings. For instance, identifying effective interventions that enhance interpersonal relationships and emotional regulation could inform the development of emotional and social education programs in schools. These programs might encompass activities and specific practices designed to foster skills such as empathy, conflict resolution, and emotional self-awareness, thereby contributing to the creation of safer and more supportive school environments.

Furthermore, understanding how different pedagogical approaches influence the socio-affective well-being of adolescents can help improve educational practices within the context of Physical Education (PE). For example, results suggesting that methodologies focused on personal and social responsibility or motor education may have positive effects on students’ interpersonal and emotional relationships could motivate educators to incorporate these approaches into their classes. This could entail changes in the structure and content of physical activities and how socio-emotional aspects of learning are addressed during PE classes. Ultimately, these improvements in pedagogical practices could contribute to greater well-being and holistic development among adolescents in the school context.

These findings provide actionable insights for educational practice. Educators should prioritize the adoption of pedagogical approaches that not only focus on physical competencies, but also integrate socio-emotional learning objectives. For example, structuring Physical Education sessions to incorporate cooperative–opposition activities can encourage empathy, emotional regulation, and effective conflict management. Additionally, policymakers should consider embedding evidence-based methodologies like TPSR and MCE into school curricula, to create more inclusive and supportive learning environments.

For instance, training programs for educators must emphasize the importance of these models, equipping teachers with the skills to facilitate meaningful interactions and to foster socio-affective growth. By adopting a systematic approach to implementing these interventions, schools can enhance not only the academic, but also the emotional and relational development of their students. Long-term studies evaluating the scalability and sustainability of these practices could further inform policy decisions, ensuring that socio-affective well-being becomes a cornerstone of contemporary education.

### 4.4. Limitations and Future Research

This review is underpinned by a comprehensive search of multiple databases and a quality analysis of the selected articles. Despite these efforts, several limitations have emerged, one of which is that the review was not required to have a control group, which may limit conclusions regarding the effectiveness of an intervention compared to a control group. In addition, the presence of a didactic pedagogy in the intervention groups was not limited; only the characteristics of the driving situations of the sessions, the presence of cooperation–opposition, were limited.

This review excluded other results from non-formal and informal education interventions, which could have provided a more complete assessment of the quality of training interventions. There was a low availability of high-quality articles and, as a result, this review did not impose a quality threshold for its studies.

Two of the review authors (US, PL) were also investigators of several included studies; however, these authors did not participate in the quality assessment of the review. This approach mitigated potential bias and ensured the objectivity and impartiality of the review process.

Finally, this systematic review has a relatively small study sample size, with only 15 studies included. As future lines of research, it could be possible to investigate the effects of the different pedagogical interventions that have been seen in the review (SEM, MCE and TPSR), within the same study. By comparing these interventions directly, researchers can gain a more comprehensive understanding of their effectiveness in promoting socio-affectiveness in cooperation–opposition situations among adolescents. This approach would provide valuable insights into the relative strengths and weaknesses of each intervention, allowing educators and policymakers to make more informed decisions about which strategies to implement in educational settings.

## 5. Conclusions

Taken together, the results of this systematic review emphasize the critical role of Physical Education (PE) in fostering students’ comprehensive development. PE provides a unique environment, where students’ emotional and relational dimensions can be nurtured alongside physical and cognitive growth. Recognizing and addressing students’ emotions within PE settings is essential for creating a positive and inclusive learning atmosphere that enhances both individual and group well-being. Moreover, fostering healthy, collaborative relationships is fundamental to promoting socio-affective and socio-physical development.

The comparison of different pedagogical models—such as Teaching Personal and Social Responsibility (TPSR), Motor Conduct Education (MCE), and the Sports Education Model (SEM)—highlights the importance of adopting evidence-based strategies to improve educational outcomes and student satisfaction. Implementing these methodologies in PE not only supports academic objectives, but also addresses the emotional and social needs of adolescents, contributing to their overall well-being. Educators should prioritize pedagogical approaches that integrate socio-emotional learning into PE curricula, leveraging cooperative–opposition activities to build empathy, resilience, and effective conflict resolution skills among students. Schools should provide targeted professional development for PE teachers to equip them with the tools needed to implement these evidence-based models effectively.

Policymakers should consider embedding these methodologies into national and regional education policies, to ensure their widespread adoption. Investing in longitudinal studies to evaluate the sustainability and scalability of these interventions is essential. Such research can provide robust evidence on the long-term impacts of these practices on students’ socio-affective development and their preparedness for future social interactions. By systematically integrating these approaches into educational systems, schools can create safer, more inclusive, and emotionally supportive environments. This holistic perspective not only enhances immediate educational outcomes, but also contributes to the development of well-rounded, socially adept individuals, equipped to navigate the complexities of modern society.

## Figures and Tables

**Table 1:** Characteristics of the studies and reported effects of the interventions.

Study, Country	Design	Time Points		Experimental Condition					Control Condition		Relational Dimension			Emotional Dimension	
			N Total	N Pre	N Post	Sessions	Measure	Effect	Intervention	Educational Pedagogy	N Pre	N Post	Educational Pedagogy	Measure	Effect
Cecchini et al. (2003) [44] Spain	QExp (prospective, comparative measure)	Pre, Post	142	72	7	10	-	-	Futsal	TPSR	70	70	No intervention	CASC-Q; EAF; OFC	It improves ethical and prosocial behaviors
Durán et al. (2014) [48] Spain	QExp (prospective, one-group prior comparison)	Pre, post, every session	220	220	220	4	GES	Positive emotions have more intensity	Motor games	Not specific	-	-	-	-	-
Falcón et al. (2020) [49] Spain	QExp (prospective, one-group prior comparison)	Pre, post, every session	67	67	67	8	GES	Positive emotions have more intensity	Motor games	Not specific	-	-	-	-	-
Fernández-Rio and Casey (2020) [50] Spain and UK	QExp (prospective, comparative measure)	Pre, post, every session	90	48	48	12 + 12	-	-	Football + basketball	SEM	42	42	TM-DI	CL-Q	Interpersonal skills increase
Haoran et al. (2023) [51] China	QExp (prospective, comparative measure)	Pre, Post	87	42	42	36	-	-	Basketball with competition phase	Not specific	45	45	Sports without a competition phase	FIAT-Q; SAICA	In EG interpersonal relationships, self-identity and social adjustment improve significantly
Luna et al. (2020) [52] Spain	RCT (prospective)	Pre, post, every session	158	114	106	16	-	-	Sport of split teams or net (Polskie ringo)	SEM	44	44	TM-DI	AMSC-Q	Improvements in prosocial behavior and cooperation
Luna et al. (2021) [53] Spain	QExp (prospective, comparative measure)	Pre, post, every session	170	87	77	18	-	-	Sport of split teams or net (Polskie ringo)	SEM	83	71	TM-DI	IAMI-R	It improves the interpersonal and intrapersonal relationship in the EG
Martins et al. (2022) [54] Portugal	QExp (prospective, comparative measure)	Pre, Post	53	27	27	24	-	-	Football	TPSR	26	26	Marked in the teaching schedule	PSR	In the EG, the variables related to personal and social responsibility improve significantly
Molina et al. (2021) [55] Spain	QExp (prospective, one-group prior comparison)	Pre, post, every session	26	26	26	6	GES	Positive emotions have more intensity	Motor games	Not specific	-	-	none	-	-
Mujica and Jiménez (2021) [56] Spain	QExp (prospective, one-group prior comparison)	every session	44	44	44	38	semi-structured interviews; personal diaries	Positive emotions in grouping by pairs	Basketball	Not specific	-	-	none	-	-
Mujica (2021) [57] Spain	QExp (prospective, one-group prior comparison)	every session	44	44	44	38	semi-structured interview; personal diaries	Negative emotions in traditional pedagogies	Basketball	Not specific	-	-	none	-	-
Pic et al. (2019) [58] Spain and Colombia	QExp (prospective, one-group prior comparison)	every session	183	183	183	1	GES-II; SHL MQ	Positive emotions have more intensity	Basketball	Not specific	-	-	none	-	-
Rillo-Albert, Lavega-Burgués, et al. (2021) [33] Spain	QExp (prospective, one-group prior comparison)	Pre, post, every session	319	319	287	7	PeerMCYSQ	Ratings on the atmosphere of the task increase	Traditional sporting games	MCE	-	-	none	PeerMCYSQ	Motor conflicts decrease
Rillo-Albert, Sáez de Ocáriz, Lavega, et al. (2021) [13] Spain	QExp (prospective, one-group prior comparison)	before, after	287	287	287	7	PeerMCYSQ	Ratings on the atmosphere of the task increase	Traditional sporting games	MCE	-	-	none	PeerMCYSQ	Interpersonal relationships improve and motor conflicts decrease
Rillo-Albert, Sáez de Ocáriz, Costes, et al. (2021) [12] Spain	QExp (prospective, one-group prior comparison)	Pre, post, three moments	222	222	222	7	GES-II	Positive emotions have more intensity	Traditional sporting games	MCE	-	-	none	MCQ	Motor conflicts decrease

*Note.* AMSC-Q: Adolescent Multidimensional Social Competence Questionnaire; CASC-Q: Child and Adolescent Self-Control Questionnaire; CL-Q: Cooperative Learning Questionnaire; EAF: Fairplay Attitudes Scale; GES: Games and Emotions Scale; GES-II: Games and Emotion Scale II; IAMI-R: Multiple Intelligences Self-Efficacy Inventory; FIAT-Q: Interpersonal Relationship Scale; MCE: Motor Conduct Education; MCQ: Motor Conflict Questionnaire; OFC: Observation of Fairplay Conducts; PeerMCYSQ: Motivational Climate of Peers in Sport; PSR: Personal and Social Responsibility Questionnaire; QExp: Quasi-experimental; RCT: Randomized study; SAICA: Social Adjustment Scale for Children and Adolescents; SEM: Sports Education Model; SHL MQ: Motivational Questionnaire; TM-DI: Traditional Direct Instruction Model; TPSR: Teaching of Personal and Social Responsibility.

## Data Availability

The data presented in this study are available on request from the corresponding author due to ethics requirements.

## Supplementary Materials

The following supporting information can be downloaded at: https://www.mdpi.com/article/10.3390/children12010015/s1, Table S1: PRISMA Checklist.
